# Feasibility of multi-parametric magnetic resonance imaging combined with machine learning in the assessment of necrosis of osteosarcoma after neoadjuvant chemotherapy: a preliminary study

**DOI:** 10.1186/s12885-020-06825-1

**Published:** 2020-04-15

**Authors:** Bingsheng Huang, Jifei Wang, Meili Sun, Xin Chen, Danyang Xu, Zi-Ping Li, Jinting Ma, Shi-Ting Feng, Zhenhua Gao

**Affiliations:** 1 Medical AI Lab, School of Biomedical Engineering, Health Science Centre, Shenzhen University, Shenzhen, China; 2 Shenzhen University General Hospital Clinical Research Centre for Neurological Diseases, Shenzhen, China; 3 Department of Radiology, the First Affiliated Hospital, Sun Yat-Sen University, Guangzhou, People’s Republic of China; 4 Department of Medical Imaging and Interventional Radiology, Sun Yat-Sen University Cancer Centre, State Key Laboratory of Oncology in South China, Collaborative Innovation Centre for Cancer Medicine, Guangzhou, People’s Republic of China; 5 National-Regional Key Technology Engineering Laboratory for Medical Ultrasound, Guangdong Key Laboratory for Biomedical Measurements and Ultrasound Imaging, School of Medicine, Shenzhen University, Shenzhen, China

**Keywords:** Osteosarcoma, Random forest, MRI, Neoadjuvant chemotherapy

## Abstract

**Background:**

Response evaluation of neoadjuvant chemotherapy (NACT) in patients with osteosarcoma is significant for the termination of ineffective treatment, the development of postoperative chemotherapy regimens, and the prediction of prognosis. However, histological response and tumour necrosis rate can currently be evaluated only in resected specimens after NACT. A preoperatively accurate, noninvasive, and reproducible method of response assessment to NACT is required. In this study, the value of multi-parametric magnetic resonance imaging (MRI) combined with machine learning for assessment of tumour necrosis after NACT for osteosarcoma was investigated.

**Methods:**

Twelve patients with primary osteosarcoma of limbs underwent NACT and received MRI examination before surgery. Postoperative tumour specimens were made corresponding to the transverse image of MRI. One hundred and two tissue samples were obtained and pathologically divided into tumour survival areas (non-cartilaginous and cartilaginous tumour viable areas) and tumour-nonviable areas (non-cartilaginous tumour necrosis areas, post-necrotic tumour collagen areas, and tumour necrotic cystic/haemorrhagic and secondary aneurismal bone cyst areas). The MRI parameters, including standardised apparent diffusion coefficient (ADC) values, signal intensity values of T2-weighted imaging (T2WI) and subtract-enhanced T1-weighted imaging (ST1WI) were used to train machine learning models based on the random forest algorithm. Three classification tasks of distinguishing tumour survival, non-cartilaginous tumour survival, and cartilaginous tumour survival from tumour nonviable were evaluated by five-fold cross-validation.

**Results:**

For distinguishing non-cartilaginous tumour survival from tumour nonviable, the classifier constructed with ADC achieved an AUC of 0.93, while the classifier with multi-parametric MRI improved to 0.97 (*P* = 0.0933). For distinguishing tumour survival from tumour nonviable, the classifier with ADC achieved an AUC of 0.83, while the classifier with multi-parametric MRI improved to 0.90 (*P* < 0.05). For distinguishing cartilaginous tumour survival from tumour nonviable, the classifier with ADC achieved an AUC of 0.61, while the classifier with multi-parametric MRI parameters improved to 0.81(*P* < 0.05).

**Conclusions:**

The combination of multi-parametric MRI and machine learning significantly improved the discriminating ability of viable cartilaginous tumour components. Our study suggests that this method may provide an objective and accurate basis for NACT response evaluation in osteosarcoma.

## Background

Osteosarcoma is the most common primary malignant bone tumour, which tends to occur in children and adolescents, and is one of the main causes of cancer death and disability in these age groups worldwide [1]. Neoadjuvant chemotherapy (NACT) has significantly improved the therapeutic effectiveness in osteosarcoma, and increased the 5-year survival rate, becoming the most critical treatment outside surgery [2]. However, the significant heterogeneity of osteosarcoma may lead to inconsistencies in treatment outcomes among patients. Thus, it is necessary to objectively evaluate the efficacy of NACT in these patients. NACT response evaluation allows terminating ineffective treatments, developing surgical regimens, and adjusting postoperative chemotherapy regimens to achieve personalised treatment, thereby improving the overall treatment outcomes [3]. Up to now, postoperative pathological tumour necrosis rate is the main criterion to evaluate the efficacy of NACT [4], but is invasive and cannot be used for real-time monitoring during NACT, or for guiding the choice of surgical timing and surgical plan, as it can be performed only postoperatively [5, 6]. Moreover, the method requires pathologists to conduct extensive information processing to interpret highly complex pathological images, making it cumbersome, costly, time-consuming, and subjective [7].

Magnetic resonance imaging (MRI) has become the most important diagnostic method for the local staging of primary bone tumours and the detection of postoperative tumour recurrence [8]. Diffusion weighted imaging (DWI) is a functional MRI method capable of reflecting the movement of water molecules in living tissues and has been used for initial assessment of osteosarcoma [9] and monitoring of chemotherapy efficacy [10]. The apparent diffusion coefficient (ADC) is the diffusion coefficient value of the biological tissue measured by DWI, and reflects the process of tumour cell growth and decline in vivo. ADC can be used to judge the degree of diffusion of water molecules and predict the curative effect before the tumour morphological changes [11, 12]. Studies have shown that an increase in ADC after NACT is associated with a favourable histological response and indicated that ADC can be used as an important parameter to detect the efficacy of NACT [13, 14]. However, some other studies have not found a significant association between ADC and tumour necrosis [15, 16]. We have previously demonstrated by statistical methods that there are no significant differences in ADC values among the cartilaginous tumour survival areas, the post-necrotic collagenised areas, and the tumour necrosis cystic/haemorrhagic areas [17], suggesting that ADC could not be used to assess the necrosis of chondroblastic osteosarcoma. Therefore, the reliability of the ADC value from DWI for NACT response assessment is still uncertain.

Machine learning has been used in the study of cancer, including breast, lung, and colon cancer, showing great diagnostic and prognostic potential [18]. However, there is still a lack of studies on NACT response assessment in osteosarcoma using machine learning applied to MRI data. In this study, we applied a machine learning method to investigate the role of multi-parametric MRI (mpMRI) in the assessment of post-NACT necrosis in osteosarcoma. We hypothesised that mpMRI combined with machine learning could improve the ability to discriminate tumour necrosis from tumour survival after NACT, and, in addition, that the combination of mpMRI and machine learning would allow noninvasive, real-time response evaluation of chemotherapy before surgery.

## Methods

This study was conducted with the approval of the Ethics Committee of the First Affiliated Hospital of Sun Yat-Sen University. Written informed consent was obtained from the patients or their parents before MRI.

### Patients and treatments

We prospectively recruited 12 patients (7 males and 5 females, mean age 14.6 ± 4.8 years) with primary osteosarcoma who were admitted to the First Affiliated Hospital of Sun Yat-Sen University from August 2011 to March 2012. Eight patients had primary osteosarcoma of the distal femur and 4 had primary osteosarcoma of the proximal tibia. The histological types included osteoblastic (*n* = 7), chondroblastic (*n* = 4), and fibroblastic (*n* = 1) osteosarcoma.

All patients received four cycles of NACT including high-dose methotrexate, pirarubicin, and ifosfamide with or without cisplatinum. Limb-salvage surgery was performed 3 weeks after chemotherapy. Routine MRI examination was performed within 3 days before surgery.

### MRI protocols

The MRI data of all patients were acquired using an extremity coil on a Siemens Magnetom Trio 3.0 T whole-body magnetic resonance scanner (Magnetom Trio, Syngo MR 2006 T, Siemens Medical Solution, Forchheim, Germany) at Department of Radiology of the First Affiliated Hospital of Sun Yat-Sen University.

T1-weighted imaging (T1WI) scanning adopted the axial spin-echo sequence with repeat time (TR) = 659 ms and echo time (TE) = 11 ms. T2-weighted imaging (T2WI) scanning adopted coronal, sagittal, and axial fast spin-echo sequences with or without fat suppression (TR = 4660 ms, TE = 96 ms). Axial DWI was performed using the single-shot spin-echo echo-planar imaging sequence with the following scan parameters: TR = 3200 ms, TE = 82 ms, echo-planar imaging (EPI) factor = 3, b-values = 0, 800 s/mm^2^. Finally, we performed a delayed enhanced scan with the same parameters as the axial non-enhanced T1WI sequence, and the subtracted images were automatically generated.

All the axial plane scans were perpendicular to the longitudinal axis of the body and parallel to the tibial plateau. The field of view and the centre of the layer were consistent (with the largest cross-section of the tumour as the centre of the layer), with slice thickness = 5 mm and interslice gap = 1 mm.

### Sampling of gross specimen sections and grouping of the sampling areas

The resected gross specimens from limb-salvage surgery were fixed in 10% buffered formaldehyde solution, and sectioned to axial slices with a thickness of 5 mm corresponding to the axial MRI layers. Section-by-section coregistration was performed between MRI and the specimens by a radiologist and an experienced musculoskeletal pathologist to select 6–10 well-matched specimen sections from each patient. Rectangular tumour tissue samples ranging from 10 × 15 mm to 15 × 20 mm were drawn on these specimen sections corresponding to the homogeneous signal intensity areas on T1WI, T2WI, and DWI. Depending on the size of tumour, 9 to 24 sampling areas were selected from each patient, and a total of 127 tissue samples were obtained from the 12 resected specimens. These tissue samples were fixed, decalcified, dehydrated, embedded with paraffin, sectioned, and stained with hematoxylin and eosin (H&E).

**Fig. 1:**
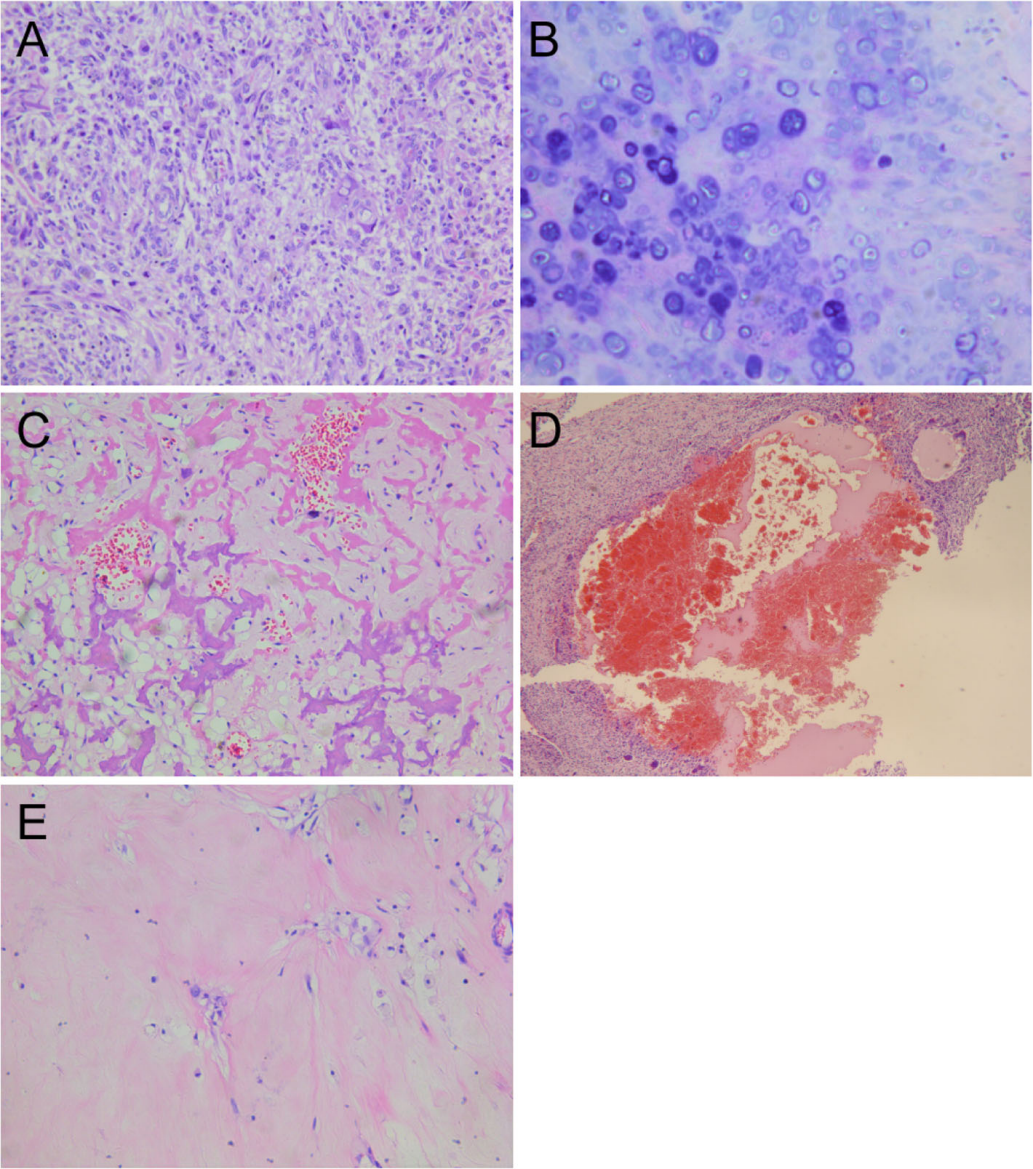
Pathological manifestations of osteosarcoma after NACT. The tissue samples were classified as five types microscopically including (**a**) non-cartilaginous tumor viable areas, (**b**) cartilaginous tumor viable areas, (**c**) non-cartilaginous tumor necrotic areas, (**d**) blood space areas and (**e**) tumor post-necrotic collagenized areas. (original magnification of a, c, e × 200, b × 400, d × 50; H&E stain)

In this study, microscopically viable sarcomatous cells, tumour osteoid, tumour bone, viable chondrosarcomatous cells with cartilaginous matrix, sarcomatous cells necrosis, post-necrotic collagen, liquefactive necrosis, blood spaces, and secondary aneurismal bone cysts (ABC) were recorded for all tissue samples by pathologists blinded to the MRI findings. Areas with tumour cell necrosis less than 10% were defined as the tumour viable areas, while areas with tumour cell necrosis greater than or equal to 90% were defined as the tumour necrotic areas. Areas with tumour cartilage greater than 50% were defined as the cartilaginous tumour, while areas with tumour cartilage less than or equal to 50% were defined as non-cartilaginous tumour. Thus, all the tissue samples were classified as non-cartilaginous tumour viable areas, cartilaginous tumour viable areas, non-cartilaginous tumour necrotic areas, tumour necrosis cystic/haemorrhagic and secondary ABC areas, and tumour post-necrotic collagenised areas. Among them, the non-cartilaginous viable tumour areas and cartilaginous viable tumour areas belong to the survival areas of tumour, while the non-cartilaginous tumour necrosis areas, collagen areas after tumour necrosis, tumour necrosis cystic/haemorrhagic and secondary ABC areas are classified as the nonviable areas of tumour [17] (Fig. 1).

### Classification with machine learning

**Fig. 2:**
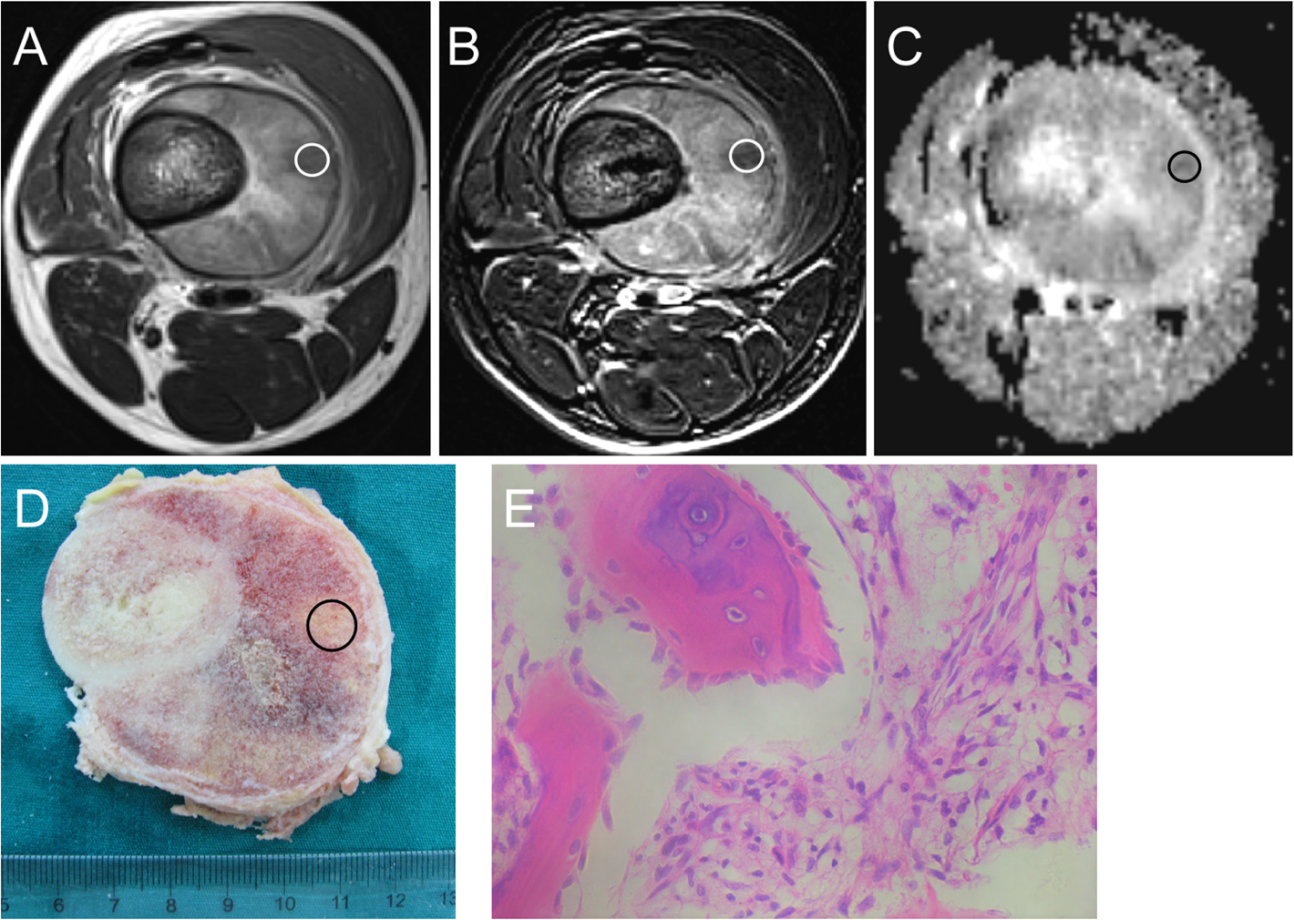
Fibroblastic osteosarcoma of distal femur in a 15-year-old boy. Using (**a**) axial T2W and (**b**) subtractedenhancedT1W images as reference, circular ROI was placed on the (**c**) ADC map inside the circular tissue sampling region of the (b) corresponding gross specimen section. Microscopically viable non-cartilaginous tumor was seen on the photomicrograph of the histological specimen (**e**) (original magnification,×400; H&E stain)

Taking axial T1WI and T2WI as reference, circular or oval regions of interest (ROIs) were placed on T2WI, subtract-enhanced T1WI (ST1WI), and the ADC maps which were coregistered to the histological sampling areas; this was performed jointly by two experienced radiologists (MLS and ZHG). The size of ROIs was in the range of 50–250 mm^2^ (Fig. 2). The average MRI parameters on the ROIs, namely ADC and the signal intensity of T2WI and ST1WI, were measured. We divided the above MRI parameters by the respective signal intensity of normal muscle to obtain the corresponding standardised values, namely rADC, rT2WI, and rST1WI.

Our previous studies have demonstrated by statistical methods that the differences in ADC values among the cartilaginous tumour survival areas, the post-necrotic collagenised areas, and the tumour necrosis cystic/haemorrhagic and secondary ABC areas were in significant [17]. Thus, in this study, we performed three classification tasks using a supervised machine learning method based on the random forest (RF) algorithm: distinguishing tumour survival from tumour nonviable; distinguishing non-cartilaginous tumour survival from tumour nonviable; and distinguishing cartilaginous tumour survival from tumour nonviable. We performed the training and testing by using the Python scikit-learn learning library (https://scikit-learn.org/stable/). The models were constructed with rADC values only, or with all the above normalised parameters, for comparisons between different feature inputs. Performance of the models was evaluated by 5-fold cross-validation. We calculated the receiver operating characteristic (ROC) curve and the area under the ROC curve (AUC) to evaluate the classification performance [19]. Using the optimal threshold determined by the ROC curve, we also calculated sensitivity, specificity and accuracy as

$$ {\displaystyle \begin{array}{l} sensitivity=\frac{\mathrm{TP}}{\mathrm{TP}+\mathrm{FN}}\\ {} specificity=\frac{TN}{TN+ FP}\\ {} accuracy=\frac{TP+ TN}{TP+ TN+ FP+ FN}\end{array}} $$

where, TP (true positive) represents the number of samples correctly predicted as positive, TN (true negative) represents the number of samples correctly predicted to be negative, FN (false negative) represents the number of samples incorrectly predicted to be negative, and FP (false positive) represents the number of samples incorrectly predicted to be positive.

## Results

### Clinical characteristics of patients

A total of 12 patients with osteosarcoma were enrolled in this study, and the baseline clinical characteristics are shown in Table 1.

**Table 1:** Summary of the clinical and pathological characteristics of the osteosarcoma patients enrolled in this study

Characteristics	Summary
Age	
Age Range	6–25 years
Mean Age	14.6 ± 4.8 years
Gender	
Male	7 (58.3%)
Female	5 (41.7%)
Primary Tumour Site	
Distal Femur	8 (66.7%)
Proximal Tibia	4 (33.3%)
Pathological Subtypes	
Osteoblastic	7 (58.3%)
Chondroblastic	4 (33.3%)
Fibroblastic	1 (8.4%)

### Pathological manifestations of osteosarcoma after NACT

The number and proportion of samples of the aforementioned five histopathological types of osteosarcoma after NACT are shown in Table 2.

**Table 2:** Number and proportion of samples in the different pathological types of osteosarcoma after NACT

Pathological types	Number of samples(%)
Non-cartilaginous tumour survival	38 (37.3%)
Cartilaginous tumour survival	14 (13.7%)
Non-cartilaginous tumour necrosis	25 (24.5%)
Tumour necrotic cystic/haemorrhagic and secondary ABC	14 (13.7%)
Post-necrotic collagen	11 (10.8%)

Among the 127 tissue samples, there were 102 homogeneous histological areas in 12 patients, including 38 of non-cartilaginous viable tumour, 25 of non-cartilaginous tumour necrosis, 14 of cartilaginous viable tumour, 14 of tumour necrotic cystic/haemorrhagic and secondary ABC, and 11 of post-necrotic collagen. In order to avoid inaccurate calculation of MRI parameters due to tissue heterogeneity, the remaining 25 heterogeneous histological areas, characterised by partial necrosis (10% < necrosis < 90%), were excluded from the analysis.

### Machine learning based classification

Table 3 shows the mean and standard deviation of the standardised average MRI parameters (rADC, rT2WI and rST1WI) on the ROIs for the aforementioned five histopathological types. Table 4 shows the classification results (using the models based on rADC alone and the combination of rADC, rT2WI, and rST1WI) in terms of accuracy, specificity, sensitivity, and AUC.

**Table 3:** MRI parameters in different pathological tissues

Pathological type	rADC	rT2WI	rST1WI
Non-cartilaginous tumour survival	0.94 ± 0.16	3.81 ± 1.84	3.36 ± 1.06
Cartilaginous tumour survival	1.53 ± 0.13	5.86 ± 1.54	3.77 ± 2.58
Non-cartilaginous tumour necrosis	1.35 ± 0.12	3.65 ± 1.50	3.12 ± 1.24
Tumour necrotic cystic/haemorrhagic and secondary ABC	1.76 ± 0.21	8.45 ± 5.09	2.32 ± 1.16
Post-necrotic collagen	1.82 ± 0.13	5.72 ± 1.97	2.85 ± 1.02

**Table 4:** Classification results using the random forest classifier

Classification Task	Features	Sen (%) [95% CI]	Spe (%) [95% CI]	Acc (%) [95% CI]	AUC [95% CI]	*p*-Value
TS vs. TN	rADC	82 [71 93]	69 [56 82]	75 [67 83]	0.83 [0.66 0.85]	0.0473
	rADC, rT2WI, rST1WI	94 [87101]	78 [67 89]	85 [78 92]	0.90 [0.78 0.93]	
CTS vs. TN	rADC	68 [55 81]	57 [31 83]	66 [54 78]	0.61 [0.46 0.80]	0.0153
	rADC, rT2WI, rST1WI	66 [53 79]	92 [78106]	71 [60 z 82]	0.81 [0.68 0.91]	
NCTS vs. TN	rADC	88 [79 97]	89 [79 99]	89 [82 96]	0.93 [0.81 0.96]	0.0933
	rADC, rT2WI, rST1WI	96 [91101]	92 [83101]	94 [89 99]	0.97 [0.88 1.00]	

*TS* tumour survival, *CTS* cartilaginous tumour survival, *NCTS* non-cartilaginous tumour survival, *TN* tumour nonviable, *Sen* sensitivity, *Spe* specificity, *Acc* accuracy; the *P* value represents the significance of the statistical comparisons of the AUCs of the different RF models (constructed with rADC vs. constructed with rADC, rT2WI, and rST1WI)

**Fig. 3:**
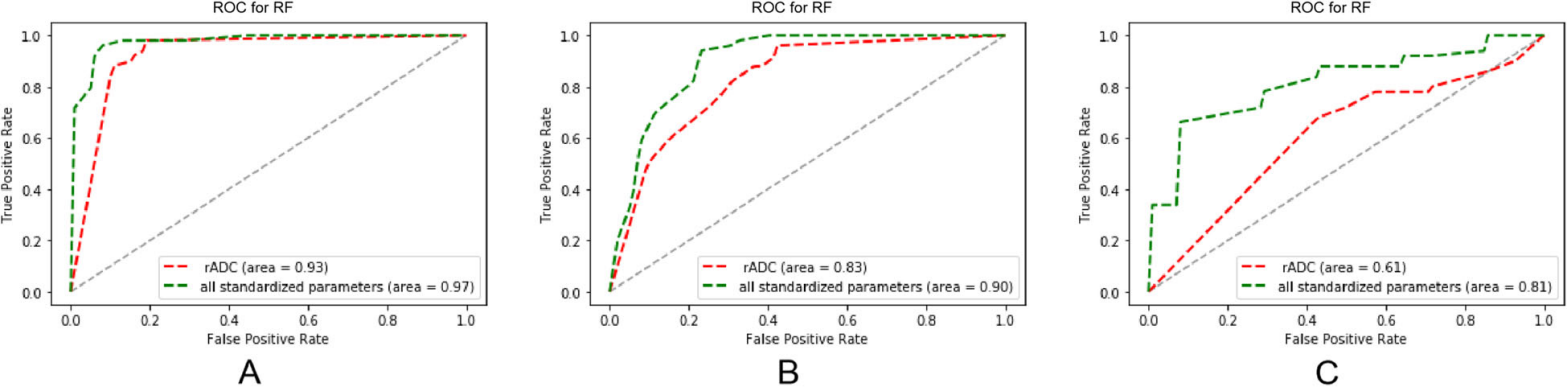
**ROC curves** of the RF classifiers. The curves in red are the ROC curves for the classification model using rADC as their only feature, while the green curves are those of the classification model using rADC, rT2WI, and rST1WI signal intensity values. **a**) ROC curves for the task of distinguishing non-cartilaginous tumour survival from tumour nonviable. **b**) ROC curves for the task of distinguishing tumour survival from tumour nonviable. **c**) ROC curves for the task of distinguishing cartilaginous tumour survival from tumour nonviable

The results showed that machine learning models constructed with rADC, rT2WI, and rST1WI signal intensity achieved better performance than those using rADC alone in all three classification tasks. For distinguishing non-cartilaginous tumour survival from tumour nonviable, using only the rADC value, we obtained a sensitivity of 88%, a specificity of 89%, an accuracy of 89%, and an AUC of 0.93. Using rADC, rT2WI, and rST1WI signal intensity values improved the sensitivity to 97%, the specificity to 92%, the accuracy to 94%, and the AUC to 0.97 (Table 3 and Fig. 3A). The combination of mpMRI features improved the classification performance, but not in a statistically significant way compared with the use of rADC alone (*P* = 0.0933).

For distinguishing tumour survival from tumour nonviable, using rADC, rT2WI, and rST1WI, we obtained a sensitivity of 94%, a specificity of 78%, an accuracy of 85%, and an AUC of 0.90. However, using rADC alone, the sensitivity was reduced to 82%, the specificity was reduced to 69%, the accuracy was reduced to 75%, and the AUC was reduced to 0.83 (Table 3 and Fig. 3b). MpMRI significantly improved the performance of the machine learning model to distinguish between tumour survival and tumour nonviable (*P* < 0.05).

For a more challenging discrimination task, namely distinguishing cartilaginous tumour survival from tumour nonviable, the classifier constructed with rADC, rT2WI, and rST1WI signal intensity values provided good classification rates (sensitivity of 66%, specificity of 92%, accuracy of 71%, and AUC of 0.81). This classifier outperformed the classifier constructed using rADC alone, with significant AUC difference of 0.20 (Table 3 and Fig. 3c; *P* < 0.05).

## Discussion

In this study, we proposed a noninvasive and accurate method that evaluates the necrosis of osteosarcoma after NACT by combining mpMRI with machine learning. The results showed that our method could distinguish tumour necrosis and tumour survival more accurately, compared with a single MRI parameter.

In distinguishing non-cartilaginous tumour survival from tumour nonviable, the machine learning based classification with rADC alone achieved an AUC of 0.93In our previous study, we have reported that the mean ADC values in non-cartilaginous tumour viable areas were significantly lower than those in non-cartilaginous tumour necrotic areas and cystic/haemorrhagic necrotic areas [17]. Hypercellularity, intact cellular membranes, and reduced extracellular volume of viable non-cartilaginous tumour cells result in lower diffusion than in necrotic non-cartilaginous tumour cells. In cystic areas of liquefied necrosis, blood space, or secondary ABC, free diffusion of water molecules leads to increased ADC. These phenomena lead to a significant difference in ADC values between non-cartilaginous tumour viable areas and tumour nonviable areas, so that the ADC value can be used as an effective parameter to distinguish non-cartilaginous tumour survival and tumour nonviable areas. Other studies have also demonstrated a significant increase in ADC value after NACT, and shown that the mean ADC value of the tumour is a predictor of treatment outcomes [9]. Wang et al. [20] found that the tumour ADC after NACT was higher in osteosarcoma patients with good response than in those with poor response, and furthermore, there was a positive correlation between overall tumour ADC and tumour necrosis rate after chemotherapy. Compared with standard statistical models, machine learning models are more flexible, discriminative, and able to capture higher-order interactions between data. Therefore, machine learning combined with different parameters can effectively make use of complementary information between mpMRI, resulting in better predictions.

The results of this study indicate that ADC has limited ability to discriminate between cartilaginous tumour survival and tumour nonviable using machine learning techniques, while mpMRI significantly improved the discriminatory ability in this task. Our previous study demonstrated that cartilaginous tumour survival areas have high ADC values, indistinguishable from post-necrotic collagenic tissue or cystic/haemorrhagic necrosis [17]. The high ADC values in cartilaginous tumour viable areas may be explained by the sparse tumour cells in the myxoid matrix of cartilaginous tumour resulting in free water diffusion [21]. Water-rich extracellular cartilaginous matrix and its high permeability may also lead to increased ADC [10]. Our previous findings also suggested that histological subtypes of osteosarcoma should be taken into account when evaluating the response to chemotherapy with DWI [17]. However, analysis of the histological subtypes for NACT response assessment is cumbersome, eveninaccurat with poor reproducibility. Our method by analyzing mpMRI may simplify the assessment and achieve timely, accurate, and reproducible results. This may be explained by that contrast-enhanced subtraction TIWI and T2WI provides the information on tumour enhancement and boundary, and that machine learning can make full use of the multiple information by optimizing their combination to construct the best prediction model for the classification tasks. Similarly, Blackledge et al. [22] showed that tissue classification with supervised machine learning methods in mpMRI of soft tissue sarcomas allows the quantitative assessment of heterogeneous tissue changes after radiotherapy.

Our results also showed that mpMRI can significantly improve the ability of machine learning models to differentiate tumour necrosis from tumour survival compared with the use of ADC alone, even if living cartilage tumours component was taken into account. This may be because mpMRI provides more valuable information for the construction of machine learning models. There is evidence that multi-parametric imaging with different functional MRI parameters provides detailed information about cancer hallmarks [23], including neoangiogenesis, cellularity, tumour microenvironment, metabolite concentration, receptor status, tissue pH, and oxygenation, which cannot be obtained from one single parameter. In this study, we used three parameters, namely DWI, T2WI, and contrast-enhanced subtraction T1WI. DWI can provide information about the number of tumour cells based on quantitative values (such as ADC) [14]. T2WI represents information about water distribution and tumour extent. Contrast-enhanced MRI (CE-MRI) reflects physiological information such as tissue vascularisation, perfusion rate, capillary permeability, and extravascular extracellular space. Subtract-enhanced MRI can remove the effects of high-signal lesions on non-enhanced T1WI and truly reflects the delayed subtraction enhancement information. Torricelli et al. [24] showed that CE-MRI subtraction may be a useful technique for assessing osteosarcoma response to chemotherapy and detecting residual viable tumour tissue. Furthermore, machine learning is able to effectively integrate information from mpMRI for accurate classification of tumour survival and necrosis.

Among the limitations of this study, we should mention that we only investigated the ability of mpMRI to classify the necrosis and survival of pathological types after NACT for osteosarcoma, but did not evaluate the response or necrosis rate of patients. However, our encouraging results provide evidence that can form the basis for a subsequent assessment of response or necrosis rate. A second limitation of this study is that we investigated the ability of mpMRI parameters to discriminate between different types of tissue based only on the local region averages. Correlation between MRI and the pathological information of entire specimen slices may provide more precise information. Future studies with larger samples are needed to investigate the relationship between MRI findings and histopathological information, so as to improve the NACT response evaluation in different subtypes of osteosarcoma.

## Conclusions

In summary, machine learning methods incorporating the information of T2WI, enhanced T1WI, and ADC could effectively distinguish tumour necrosis from tumour survival, or from the necrosis of the tumour extracellular matrix/stroma, after chemotherapy. Our study suggests that machine learning applied to mpMRI may provide an objective and reliable method for NACT response evaluation in osteosarcoma.

## Data Availability

Original data and material can be available from the corresponding author if necessary.

## Abbreviations

ADC: apparent diffusion coefficient
AUC: area under the ROC curve
CE-MRI: contrast-enhanced MRI
DWI: diffusion weighted imaging
EPI: echo-planar imaging
FN: false negative
FP: false positive
mpMRI: multi-parametric MRI
MRI: magnetic resonance imaging
NACT: neoadjuvant chemotherapy
rADC: standardised apparent diffusion coefficient
R: random forest
ROC: receiver operating characteristic
ROIs: regions of interest
rT2WI: standardised T2-weighted imaging signal
rST1WI: standardised subtract-enhanced T1-weighted imaging signal
ST1WI: subtract-enhanced T1WI
T1WI: T1-weighted imaging
T2WI: T2-weighted imaging
TE: echo time
TN: true negative
TP: true positive
TR: repeat time
